# Loculated hydrocephalus: is neuroendoscopy effective and safe? A 90 patients’ case series and literature review

**DOI:** 10.1007/s00381-022-05747-6

**Published:** 2022-11-29

**Authors:** Alice Noris, Flavio Giordano, Simone Peraio, Matteo Lenge, Regina Mura, Letizia Macconi, Raffaella Barzaghi, Lorenzo Genitori

**Affiliations:** 1 Neurosurgery Unit, Department of Neurosciences, Meyer Children’s Hospital, Viale Gaetano Pieraccini, 24, 50139 Florence, Italy; 2 Radiology Department, Meyer Children’s Hospital, 50139 Florence, Italy; 3 Department of Neurosurgery and Gamma Knife Radiosurgery, IRCCS San Raffaele Scientific Institute and Vita-Salute University, 20132 Milan, Italy

**Keywords:** Complex hydrocephalus, Neuroendoscopy, Neuronavigation, Pediatric, Third ventriculostomy, Ventriculoperitoneal shunt

## Abstract

**Introduction:**

Loculated hydrocephalus is a complex condition in which different non-communicating compartments form within the ventricular system due to different etiology, mainly intraventricular hemorrhage and infection. Since the end of the twentieth century, neuroendoscopy has been explored as a therapeutic option for loculated hydrocephalus with non-univocal results.

**Methods:**

We performed a retrospective analysis of 90 patients who underwent endoscopic treatment for loculated hydrocephalus from January 1997 to January 2021 (mean age: 2 years, range 7–21). We included 37 (41.1%) children with multiloculated hydrocephalus, 37 (41.1%) with isolated lateral ventricle, 13 (14.4%) with excluded temporal horn, and 3 (3.3%) with isolated fourth ventricle. We compared our results with those available in literature.

**Results:**

A mean of 1.91 endoscopic procedure/patient were performed (only one endoscopy in 42.2% of cases). Complications of neuroendoscopy and of shunt surgeries were recorded in 17 (18.9%) and 52 (57.8%) children, respectively. Twenty-six (28.9%) children were shunt-free at the last follow-up, 47.8% have only one shunt.

**Discussion:**

The first goal of neuroendoscopy is to increase the rate of shunt-free patients but, when it is not possible, it aims at simplifying shunt system and reducing the number of surgical procedures. In our series, neuroendoscopy was able to achieve both these goals with an acceptable complication rate. Thus, our results confirmed neuroendoscopy as a valid tool in the long-term management of loculated hydrocephalus. Neuronavigation and intraoperative ultrasound could increase the success rate in cases with distorted anatomy.

## Introduction

The presence of one or more isolated compartments within the ventricular system that do not communicate and tend to enlarge despite a functioning shunt system is defined as loculated hydrocephalus [1]. Intraventricular septations or obstructions between the site of CSF production and the tip of the ventricular catheter can represent a barrier to the CSF flow and can result in an accumulation of fluid in the excluded compartments [2].

Different classification systems have been proposed. The worldwide accepted classification is the one from Spennato et al. that distinguishes four types of loculated hydrocephalus basing on anatomical appearance and site of obstruction [2]. Etiologies are different; the most relevant are insults occurring in the neonatal period, namely intraventricular hemorrhage (IVH) or neonatal infections.

Several surgical approaches have been suggested but, given the complexity of this condition, none has been considered superior, judged unsafe, or abandoned. Each treatment carries its own pros and downsides and often a combination of procedures is required. Neuroendoscopy has been increasingly explored in the last decades and is showing promising results. Its aims more at reducing the number of surgical procedures and simplifying shunt systems than at improving patients’ quality of life. In fact, the latter is more influenced by the underlying disease rather than by surgical treatment itself. In selected patients, endoscopy is able to free the patients from shunts.

## Methods

### Patients’ selection

**Fig. 1:**
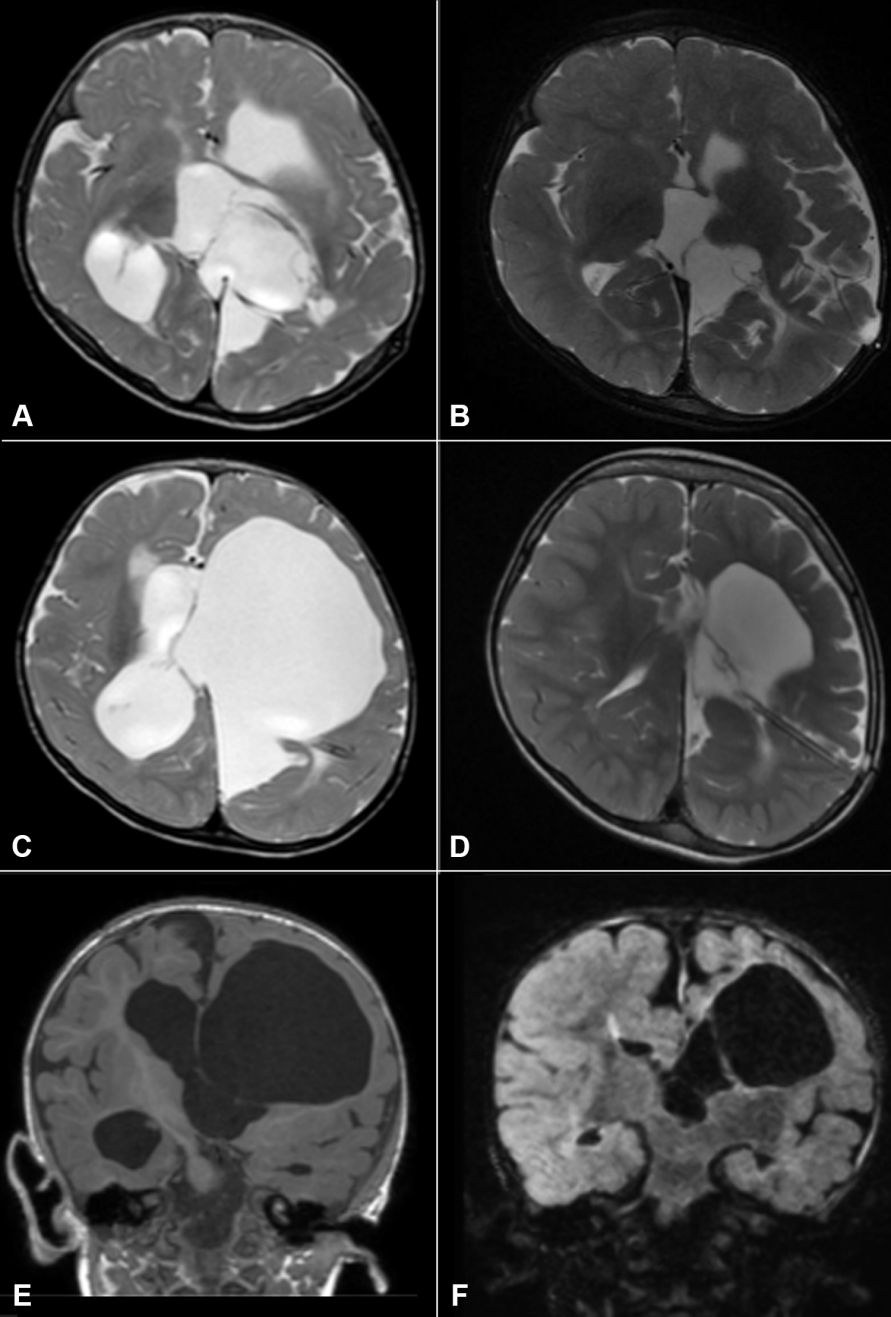
Axial T2-weighted (**A**–**D**) and coronal T1-weighted (**E**–**F**) MRI sequences of a patient with multicystic hydrocephalus. Various septation create several different compartments within the ventricular system and a significant part of brain parenchyma is lost. Form preoperative (**A**, **C**, **E**) to postoperative (**B**, **D**, **F**) images a reduction of the ventricular loculation is visible

**Fig. 2:**
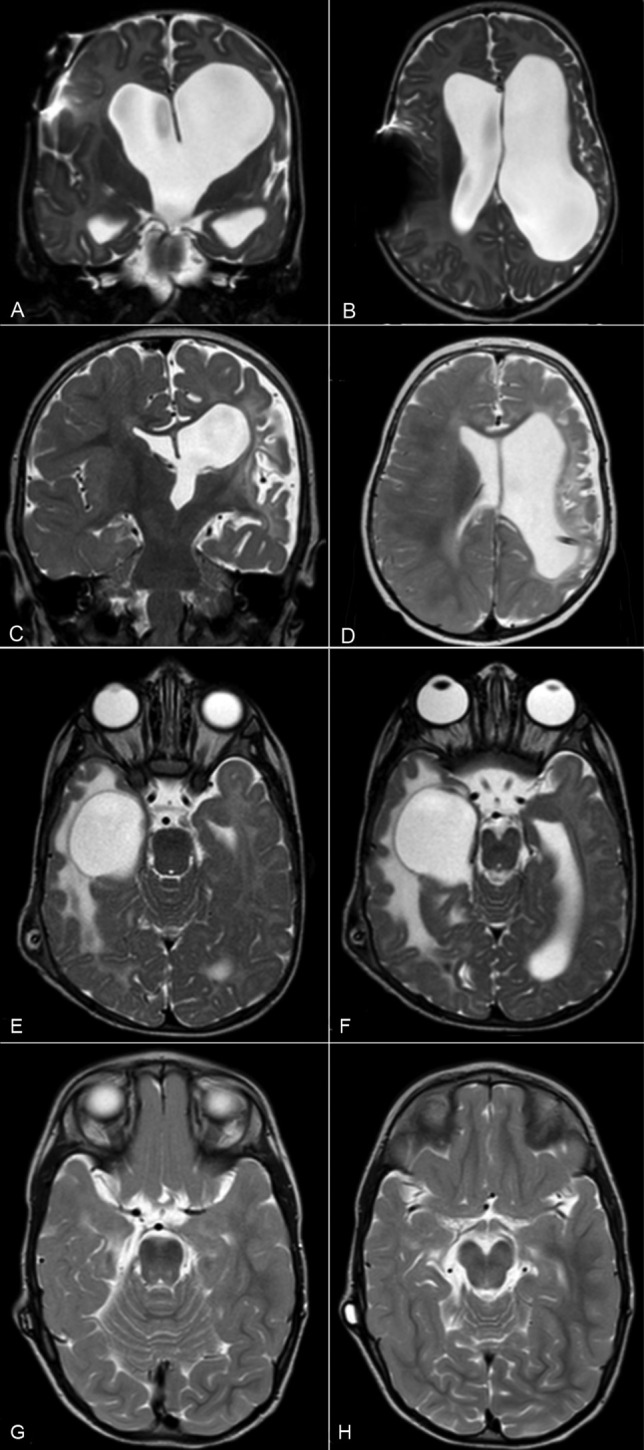
Preoperative coronal (**A**) and axial (**B**) T2-weighted MRI sequence and postoperative coronal (**C**) and axial (**D**) T2-weighted MRI sequences of a child suffering from unilateral ventricular enlargement. Preoperative axial (**E**, **F**) and postoperative axial (**G**, **H**) T2-weighted MRI sequence of a patient with entrapped temporal horn. In the postoperative images, the ventricular loculation is no longer visible

**Fig. 3:**
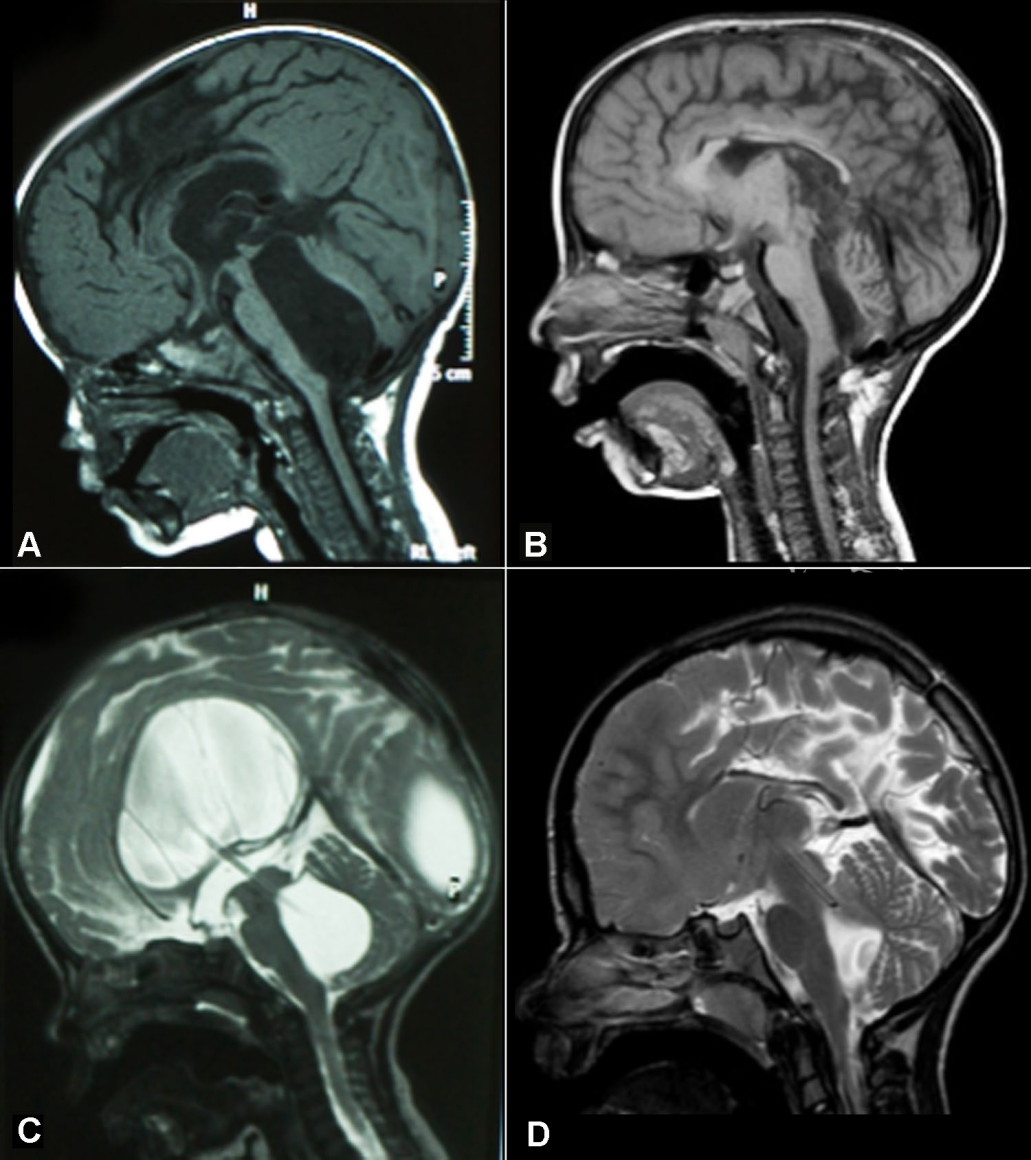
Preoperative (**A**) and postoperative (**B**) T1-weighted MRI sequences of a patient suffering from isolated fourth ventricle that underwent and endoscopic aqueductoplasty, no stent was left in the sylvian aqueduct. Preoperative (**C**) and postoperative (**D**) T2-weighted MRI sequences of a child presenting with isolated fourth ventricle that underwent and endoscopic aqueductoplasty. In this patient, a stent was placed across sylvian aqueduct

We retrospectively analyzed 90 patients who underwent endoscopic treatment of loculated hydrocephalus by the senior authors from January 1997 to January 2021. All patients presented with complex hydrocephalus and were grouped according to the classification proposed by Spennato et al. [2]: multiloculated hydrocephalus (Fig. 1), unilateral hydrocephalus (Fig. 2), entrapped temporal horn (Fig. 2), and isolated fourth ventricle (Fig. 3).

Ethics committee approval and patient consent were not required since we conducted a retrospective analysis with collection of anonymized data.

### Data collection

Patients’ medical history, data concerning endoscopic procedures, VP-shunt surgeries and other operations, and outcome have been gathered from clinical records of the database of Meyer Children’s Hospital.

Computed tomography (CT) and/or magnetic resonance imaging (MRI) were performed in all patients preoperatively to confirm the diagnosis, determine type of complex hydrocephalus, and assess site of septations and number of isolated chambers inside ventricular system. T2-weighted with flow and 3-dimensional (3D) MRI sequences revealed particularly useful in delineating loculations anatomy and helped in deciding the most suitable endoscopic procedure and in guiding the ideal surgical trajectory.

Complications for endoscopic (intraoperative and postoperative) and VP-shunt surgeries were recorded. Their management was described.

### Instrumentation and surgical technique

At least one endoscopic surgical procedure was performed in all patients. Aims of neuroendoscopy included hydrocephalus resolution, loculations fenestration, or reduction of the number of shunts placement. To reach these goals, we tried to connect the isolated chambers to the chamber drained by a shunt or with a functioning CSF pathway. Endoscopic procedures performed included third ventriculostomy (ETV), membrane fenestration, septum pellucidotomy, foraminoplasty, choroid plexus coagulation, and acqueductoplasty + /− stent insertion. We often combined more than one procedure.

A pediatric rigid endoscope (Genitori-Storz^®^) with 30° optics was used in all cases. Monopolar cautery or thulium laser (Revolix^®^) were utilized to open intraventricular septations. Fenestrations were enlarged utilizing scissors, forceps, and a 2Fr Fogarty balloon. The latter was also used for aqueductoplasty. During this procedure, if deemed necessary, a stent was left crossing the aqueduct. The site of the burr hole was chosen based on the easiest trajectory to allow cyst wall fenestration in the thinnest and most avascular regions, easier manipulation, best angle of vision, and shortest pathway through brain parenchyma.

Complex shunt revisions were assisted with endoscopic guidance or neuronavigation. The first allowed a direct visualization of the catheter and of possible septations and helped the correct placement of the new ventricular catheter. The second was particularly useful for catheter insertion in small or very distorted anatomy, where anatomical landmarks proved inadequate to provide the appropriate surgical trajectory.

### Statistical analysis

Continuous data are expressed as mean and categorical variables as frequencies/percentages. One-way ANOVA test was used to determine whether differences between means of independent groups were statistically significant. A probability value < 0.05 was considered indicative of statistical significance.

## Results

Ninety patients were included in the study. All but one patient were under 18 years of age. A total of 172 endoscopic procedures have been performed. Data regarding patients’ demographics, hydrocephalus classification, and etiologies are summarized in Table 1.

**Table 1:** Summary of relevant data regarding patients’ demographics, hydrocephalus classification, etiologies, and shunt surgeries before the first endoscopic procedure

**Demographics, classification, and etiologies**	**Values**
Sex (M/F) − no. (%)	54 (60)/36 (40)
Age in years − mean (range, + / − SD)	2 (7–21, + / − 4.29)
Follow-up period in months − mean (range, + / − SD)	119 (1–292, + / − 76.91)
Hydrocephalus subgroups − no. (%)	
Multiloculated Unilateral lateral ventricle Entrapped temporal horn Isolated fourth ventricle	37 (41.1) 37 (41.1) 13 (14.4) 3 (3.3)
Etiology* − no. (%)	
IVH prematurity Infection Tumor Overdrainage Trauma Congenital	50 (55.6) 43 (47.8) 8 (8.9) 2 (2.2) 1 (1.1) 23 (25.6)
No. of shunt/patient (before first endoscopy) − no. (%)	
None One Two	48 (53.3%) 35 (38.9%) 7 (7.8%)
No. shunt revisions (before first endoscopy) − no. (%)	
None One Two Three Four Six	17 (40.5%) 11 (26.2%) 6 (14.3%) 4 (9.5%) 3 (7.1%) 1 (2.4%)
Mean revision rate/patient (mean + / − SD)	0.59 + / − 1.18

*In many children, more than one etiological factor is present

Table 2 summarizes data concerning endoscopic procedures. Thirty-eight (42.2%) patients underwent only one, 28 (31.1%) two, and 21 (23.3%) three endoscopic surgeries. Four, five, or six procedures in one (1.1%) child each.

**Table 2:** Summary of data concerning endoscopic procedures. Complications of neuroendoscopy were recorded in 17 (18.9%) children. They comprise intraoperative complications (i.e., bleeding and need to stop the procedure) and postoperative complications (i.e., cranial nerves deficits, cerebrospinal fluid (CSF) leak, and subdural collection)

**Endoscopic procedures’ data**	**Values**
No. of endoscopic procedures − no. (%)	
One Two Three Four Five Six	38 (42.2%) 28 (31.1%) 21 (23.3%) 1 (1.1%) 1 (1.1%) 1 (1.1%)
Complications of endoscopy − no. (%) Procedure discontinuation Intraoperative bleeding CSF leak Cranial nerves deficits (i.e., III, VI, or VII c.n.) Subdural collection	17 (18.9%) 9 (10%) 4 (4.4%) 4 (4.4%) 3 (3.3%) 2 (2.2%)
Complication requiring surgery (CSF leak) − no. (%)	3 (3.3%)
Death at last follow-up − no. (%)	0

*CSF* cerebrospinal fluid, *c.n*. cranial nerve

Complications of neuroendoscopy (intraoperative and postoperative) were recorded in 17 (18.9%) children. In 9 (10%) patients the procedure was stopped because of bleeding or because not feasible. Four (4.4%) cases had intraoperative bleeding, only in 2 of them this obliged to abort the procedure. Three (3.3%) children suffered from transient cranial nerves deficits (i.e., III, VI, or VII). Only 3 out of 4 patients with CSF leak required a new surgery. Subdural collection manifested in 2 (2.2%) patients, but it was managed conservatively. No patient died at last follow-up.

As shown in Table 3, a mean of 1.91 endoscopic procedure/patient was performed (range: 1–6/patient). In the subgroups of multiloculated hydrocephalus, unilateral hydrocephalus, entrapped temporal horn, and isolated fourth ventricle, mean number of endoscopic procedures was 2.03/patient, 1.59/patient, 2.33/patient, and 2.38/patient, respectively. The differences in those means were statistically significant (*p*-value 0.047). Mean revision rates after endoscopy were 1.59/patient, 0.92/patient, 0.33/patient, and 2.38/patient, respectively. The differences in those means were statistically significant (*p*-value 0.049). Overall mean revision rate was 1.39/patient.

**Table 3:** Summary of data regarding number of endoscopic procedures in a single patient, revision rate after the first endoscopic procedure/patient, and shunt-free rate with a distinction between different subgroups of loculated hydrocephalus

**Endoscopic and shunt surgeries’ data**	**Values**	**Values divided for subgroups of loculated hydrocephalus**			
		**ML**	**UVL**	**ETH**	**IFV**
No. of endoscopic procedures/patient − mean (range, + /− SD)	1.91 (1–6, + / − 0.99)	2.03 + / − 1.12	1.59 + / − 0.73	2.33 + / − 0.58	2.38 + / − 1.12
Mean revision rate/patient − mean + /− SD	1.39 + / − 1.83	1.59 + / − 2.07	0.92 + / − 1.32	0.33 + / − 0.58	2.38 + / − 1.12
Shunt-free rate − no./total (%)	26/90 (28.9)	9/37 (24%)	15/37 (41%)	0/3 (0%)	2/13 (15%)

*ML* multiloculated, *ULV* unilateral lateral ventricle, *ETH* entrapped temporal horn, *IFV* isolated fourth ventricle, *SD* standard deviation

A summary of data regarding shunt surgeries after the first endoscopic procedure is shown Table 4. Before first endoscopic procedure, the number of shunts implanted in a single patient was 0 in 48 (53.3%), 1 in 35 (38.9%), and 2 in 7 (7.8%) patients. Of 42 (46.7%) patients who already had a shunt implanted before endoscopy, the shunt had never been revised in 17 (40.5%) patients, 1 revision in 11 (26.2%), 2 in 6 (14.3%), 3 in 4 (9.5%), 4 in 3 (7.1%), and 6 in 1 (2.4%) patient. Mean revision rate was 0.59/patient.

**Table 4:** Summary of data regarding shunts surgeries after the first endoscopic procedure

**Data regarding shunt surgeries after the first endoscopic procedure**	**Values**
No. of shunt/patient* − no. (%) None One Two Three	25 (27.8%) 43 (47.8%) 20 (22.2%) 2 (2.2%)
No. shunt revisions – no. (%)	
None One Two Three Four Five Six Seven Eight	19 (30.1%) 16 (25.4%) 10 (15.9%) 8 (12.7%) 5 (7.9%) 3 (4.8%) 3 (4.8%) 1 (1.6%) 1 (1.6%)
Shunt-related complications – no. (%) Malfunction Infection Overdrainage CSF leak	52 (57.8%) 42 (46.7%) 17(18.9%) 9 (10%) 1 (1.1%)

*At least one VP shunt was present in 65 (72.2%) patients

After first endoscopic procedure, the number of shunts implanted in a single patient was 0 in 25 (27.8%) patients, 1 in 43 (47.8%), 2 in 20 (22.2%), and 3 in 2 (2.2%) patients. It means that one VP shunt was present in 65 patients (72.2%). Of 63 (70%) patients who had a shunt after endoscopy, it had never been revised in 19 (30.1%) patients, 1 revision in 16 (25.4%), 2 in 10 (15.9%), 3 in 8 (12.7%), 4 in 5 (7.9%), 5 in 3 (4.8%), 6 in 3 (4.8%), 7 in 1 (1.6%), and 8 revisions in 1 (1.6%) patient.

Shunt-related complications manifested in 52 (57.8%) patients. The most frequent was shunt malfunction (46.7%), which was managed with shunt revision or attempt of a new endoscopic procedure. Seventeen (18.9%) patients developed a shunt infection (treated with shunt removal, placement of external ventricular drainage, antibiotic therapy, and shunt replacement once the infection was resolved). Other complications included shunt overdrainage (10.0%), resolved with valve or ventricular catheter substitution, and CSF leak (1.1%).

In our series, 26 (28.9%) children were shunt-free at the last follow-up. Among them, 9 had a previous shunt that had been removed after endoscopy. In the subgroups of multiloculated hydrocephalus, unilateral hydrocephalus, entrapped temporal horn, and isolated fourth ventricle, mean shunt-free rates after endoscopy were 0.24, 0.41, 0, and 0.15, respectively (Table 3).

To note, differences in shunt-free rate and mean number of VP-shunt after endoscopy were not statistically significant (*p*-value 0.162 and 0.111, respectively) between subgroups of loculated hydrocephalus.

**Fig. 4:**
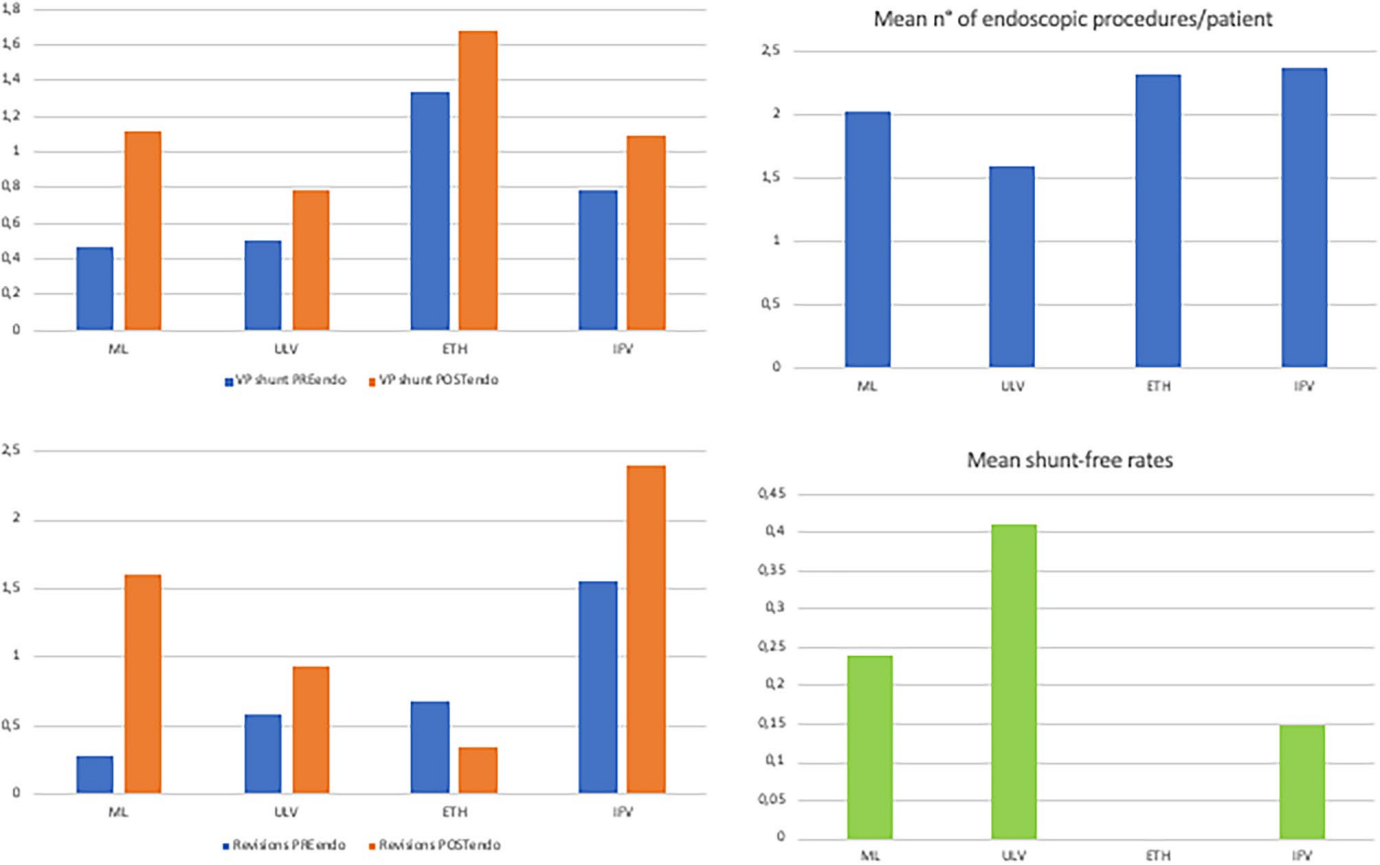
Bar graphs showing, in the subgroups of multiloculated hydrocephalus (ML), unilateral hydrocephalus confined to the lateral ventricles (ULV), entrapped temporal horn (ETH), and isolated fourth ventricle (IFV), the mean number of shunts implanted in a single patient before and after the first endoscopic procedure (**A**), the mean number of revisions in a single patient before and after the first endoscopic procedure (**B**), the mean number of endoscopic procedures/patient (**C**), and the shunt-free rate (**D**), respectively. It must be noted that the follow-up duration for many patients is long so there is a higher chance that a single patient can undergo multiple shunt revisions, even after endoscopic procedures

Figure 4 shows mean values of shunts implanted/patient before and after the first endoscopic procedure (A), of revisions/patient before and after the first endoscopic procedure (B), of endoscopic procedures/patient (C), and the shunt-free rate (D), in the subgroups of multicystic hydrocephalus.

## Discussion

### Classifications

Multiloculated and uniloculated hydrocephalus have begun to be considered separately because of their differences in etiologies, treatment strategies, risk of recurrence, and prognosis. Spennato et al. classified the five subgroups of loculated hydrocephalus and suggested treatment type based on the site of obstruction [2]. In 2012, Andresen and Juhler considered uni- and multiloculated hydrocephalus as two different variants with marked differences in outcome [3]. Their classification combined anatomical model (i.e. how many compartments are present) with consideration regarding pathophysiology and aimed at helping in the therapeutic decision-making. Instead, it separates too extensively among categories with similar prognosis and their subtypes differ from those of any other study, making comparison very difficult.

### Etiologies

In 1999, Oi et al. summarized different causes of loculated hydrocephalus indicating shunt overdrainage and post-inflammatory response as the most probable etiologies [4]. Insults occurring during neonatal period, namely IVH (particularly in premature and low-weight newborns) and neonatal meningitis or ventriculitis (especially from Gram-negative bacteria), represented the second most common causes. Other etiologies are shunt-related infection, chronic shunt overdrainage, direct ependymal trauma, head injury, congenital malformations, and intracranial surgery [2, 3].

In our series, IVH and infection were the main causes. IVH was the most frequent in the subgroup of isolated fourth ventricle (69.2%) and infection in multiloculated hydrocephalus (56.8%). Those are also the subgroups with the higher rates of shunt necessity or revision and with the worst outcomes.

### Diagnosis

CT and MRI are the most widely used techniques for diagnosis, but plain images may fail in accurately defining the communications between different intraventricular compartments. As previously stated by other authors [2, 5, 6], we believe that Constructive Interference in Steady State (CISS) and 3D T2-weighted MRI sequences and flow studies are the best sequences to visualize septations and CSF dynamics. They also help in identifying site of burr hole placement and endoscopic trajectory and in monitoring patients over time.

### Treatment

For loculated hydrocephalus the conventional treatment strategy consisted in shunt insertions of multiple multi perforated ventricular catheters in all isolated compartments. Traditional shunt treatment was associated with increased rate of infection and mechanical failure, resulting in high morbidity and mortality [1].

An alternative therapeutic strategy is endoscopic surgery. Firstly reported by Powers in the 1990s [7], it was employed for treatment of hydrocephalus in the early twentieth century. However, at that time, mortality and morbidity were high and therefore it was abandoned. Subsequently, thanks to the advances in medical technology that consented to lower the complication rates, neuroendoscopic procedures have regained interest [1, 4, 5, 8]. It was proposed with the aim to restore communication between ventricular chambers, to simplify CSF-shunts, to lyse adherences and release previous ventricular catheters, and to treat CSF-shunt infections [2].

The first large series of endoscopic treatment of loculated hydrocephalus was published by Lewis et al. in 1995 [8]. Endoscopy reduced shunt revision annual rate from 3.04 to 0.25. After this presentation, neuroendoscopy became popular [6], however, randomized studies comparing treatment options for loculated hydrocephalus are lacking. Nowosławska et al. published the only study where neuroendoscopic treatment of loculated hydrocephalus was compared to conventional implantation of shunt systems (43 endoscopy/80 control group) [9]. They concluded that neuroendoscopic cases experienced a significantly higher clinical improvement, better outcome (86% vs. 60% of good outcome), fewer postoperative complications (23% vs. 66%), and reoperations (average of 1.76 vs. 7.05 operation/patient, 3.95 vs. 1.02 revisions/year) compared to shunt control group. These results were promising, but not distinguished between uni- and multiloculated hydrocephalus. Consequently, the evidence regarding only multiloculated hydrocephalus are provided only by small case series.

Other studies confirmed the finding that, following endoscopic fenestration, a reduced number of shunt revisions per year is necessary [1, 6, 10, 11]. In 2007, Spennato et al. reported a reduction from 2.07 to 0.35; single shunt or no shunt were present in 83.3% of cases [6]. In the series of El-Grandour  (31 children with uniloculated hydrocephalus) annual shunt revision rate fell from 2.7 to 0.25 [10]. In 2011, Zuccaro and Ramos retrospectively analyzed a series of 93 patients with multiloculated hydrocephalus treated by microsurgical fenestration, endoscopic fenestration, or shunting procedures [1]. They obtained hydrocephalus control in all the patients, in 61.8% with only one procedure. They concluded that the goal of treatment is to restore the communication between isolated ventricular compartments eliminating the need of shunt or, if that is not possible, simplifying the shunting system. They aimed at reducing the number of surgical procedures more than improving the patients’ quality of life. Teo et al. in 2013 described the widest series available in literature of patients with loculated hydrocephalus treated endoscopically [11]. At follow up, 72 of 147 patients had only one shunt implanted and only 11% of cases required shunt revisions.

Our shunt revision rate after endoscopy was 1.39/patient. Interestingly, the subgroups of multiloculated hydrocephalus and isolated fourth ventricle had a statistically significant higher rate (1.59/patient and 2.38/patient, respectively; p-value: 0.049).

In most cases, neuroendoscopy is considered the treatment of choice for loculated hydrocephalus but, in case of its failure, shunt placement is often required. Consequently, the second-best goal is considered to implant a simplified shunt system. Neuroendoscopic procedures appear to achieve these goals [1, 5, 10, 12]. Our series seems to confirm these findings: 75.6% patients had no shunt or only one shunt implanted after neuroendoscopy. A mean of 1.91 endoscopic procedures/patients were performed (42.2% underwent only one endoscopy). The subgroups multiloculated hydrocephalus, entrapped temporal horn, and isolated fourth ventricle had the highest rates (2.03/patient, 2.33/patient, and 2.38/patient, respectively). The differences in those means were statistically significant (*p*-value 0.047).

In literature, rates regarding the need for a shunt after the endoscopic procedure are variable and higher in series with predominance of patients affected by multiloculated hydrocephalus. Zuccaro and Ramos reported a shunt rate of 98%, Lewis et al. 79%, Peraio et al. 90%, Spennato et al. 73%, El-Ghandour 88%, Teo et al. 72%, and Schulz et al. 100% [1, 5, 6, 8, 10, 11, 13]. Our rate is 72.2% and we included 37 patients (41.1%) with multiloculated hydrocephalus.

Despite these encouraging findings, the rate of shunt-free patients is low. In our study, shunt-free rate was 28.9%, which is in line with data reported by other authors (26%, 28%, and 10% in Spennato’s, Teo’s, and Peraio’s series, respectively) [2, 7, 11]. Percentages are higher considering only uniloculated hydrocephalus: 33% and 61.3% in Lewis’ and El-Ghandour’s series, respectively [5, 10]. Fritsch and Mehdron considered only children older than 1 year of age [12]. Their shunt-free rate was 47% (follow-up period: 24 months). In our series, the mean shunt-free rates after endoscopy were higher in children affected by unilateral hydrocephalus confined to the lateral ventricles (0.41). On the contrary, no patients affected by entrapped temporal horn and only 2 of 13 children with isolated fourth ventricle (0.15) achieved shunt independence. The differences in those means were not statistically significant (*p*-value 0.162) probably because of the small sizes of the subgroups. Peraio et al. reported similar findings [5].

Given complex anatomy of multiloculated hydrocephalus, neuronavigation is useful in maintaining orientation during the operation and avoiding damaging neural structures, even though the risk of substantial shift in anatomical landmarks after fenestration of the isolated fluid compartments remains a major problem [1, 5, 13]. Possible alternatives are the use of stereotactic or ultrasound guidance to aid ventricular catheter insertion.^8^ However, in early reports, stereotaxy is associated with high recurrence rate (37.4%) [8].

Notwithstanding neuroendoscopy is considered by many authors the most valuable tool in the treatment of multiloculated hydrocephalus, some argue against it criticizing the poor visualization and the consequent inability to obtain adequate septations’ fenestration and to ensure adequate bleeding control. According to Sandberg et al. only uniloculated hydrocephalus or simpler forms of multiloculated hydrocephalus could benefit from endoscopic treatment, while multiple fenestrations in patients with complicated multiloculated hydrocephalus were most effectively achieved microscopically [14]. Lee et al. agreed with Sandberg et al. about the advantages of craniotomy but they highlighted that it is also associated with higher risk of subdural collection and shunt malfunction [15]. The advantages of endoscopy include minimal invasiveness, avoidance of brain retraction, low blood loss, faster operation time, and shorter hospital stay. They concluded that endoscopic fenestration is commonly performed as initial treatment and that open craniotomy may be useful when repeated endoscopic procedures are needed [15]. Similar findings were described by Akbari et al. [16].

All studies agree on the fact that neuroendoscopy significantly decreases the morbidity related to the repeated procedures [4]. Our endoscopy complication rate was 18.9%, Most of the complications were mild and resolved spontaneously. Only 3 children needed surgery to fix a CSF leak. Conversely, shunt surgery is characterized by a high rate of complication and of repeated procedures. In our series, shunt-related complications were registered in 57.8% of patients and almost all of them required further surgeries.

Our study includes only pediatric patients, and, to our knowledge, it has the largest population with the longest follow-up (a mean of 119 months) in literature. The series of Peraio et al. comprises only 68 patients with a lower mean follow-up (60 months) [5]. Moreover, we included a detailed literature review of the different aspects of this complex topic, proving that neuroendoscopy is a safe and effective way of dealing with multiloculated hydrocephalus.

## Conclusions

Multiloculated hydrocephalus is a complex and challenging condition, whose outcome is often extremely poor regardless the treatment type. Neuroendoscopy proved to be efficacious in reducing the number of shunts (75.6% with no or only one VP-shunt in our series) and shunt revisions in multiloculated hydrocephalus with acceptable morbidity and mortality. In many cases, it is even able to improve patients’ quality of life. For these reasons, nowadays most neurosurgeons agreed to consider neuroendoscopy a standard treatment for multiloculated hydrocephalus. Due to distorted anatomy, integrated tools (such as neuronavigation, intra-operative ultrasound, and MRI) are required to increase success rate.
